# Partitioning of Minimotifs Based on Function with Improved Prediction Accuracy

**DOI:** 10.1371/journal.pone.0012276

**Published:** 2010-08-19

**Authors:** Sanguthevar Rajasekaran, Tian Mi, Jerlin Camilus Merlin, Aaron Oommen, Patrick Gradie, Martin R. Schiller

**Affiliations:** 1 Department of Computer Science and Engineering, University of Connecticut, Storrs, Connecticut, United States of America; 2 School of Life Sciences, University of Nevada Las Vegas, Las Vegas, Nevada, United States of America; Dana-Farber Cancer Institute, United States of America

## Abstract

**Background:**

Minimotifs are short contiguous peptide sequences in proteins that are known to have a function in at least one other protein. One of the principal limitations in minimotif prediction is that false positives limit the usefulness of this approach. As a step toward resolving this problem we have built, implemented, and tested a new data-driven algorithm that reduces false-positive predictions.

**Methodology/Principal Findings:**

Certain domains and minimotifs are known to be strongly associated with a known cellular process or molecular function. Therefore, we hypothesized that by restricting minimotif predictions to those where the minimotif containing protein and target protein have a related cellular or molecular function, the prediction is more likely to be accurate. This filter was implemented in Minimotif Miner using function annotations from the Gene Ontology. We have also combined two filters that are based on entirely different principles and this combined filter has a better predictability than the individual components.

**Conclusions/Significance:**

Testing these functional filters on known and random minimotifs has revealed that they are capable of separating true motifs from false positives. In particular, for the cellular function filter, the percentage of known minimotifs that are not removed by the filter is ∼4.6 times that of random minimotifs. For the molecular function filter this ratio is ∼2.9. These results, together with the comparison with the published frequency score filter, strongly suggest that the new filters differentiate true motifs from random background with good confidence. A combination of the function filters and the frequency score filter performs better than these two individual filters.

## Introduction

Minimotifs are short contiguous peptide pieces of proteins that have a known biological function. These functions can be categorized into binding, posttranslational modification of the minimotif, and protein trafficking. While there are many known functional minimotifs, predicting a minimotif in a new protein based on a consensus sequence, position-specific scoring matrix, or other algorithms produces many false-positive predictions. This limits the usefulness of minimotif prediction programs such as Minimotif Miner (MnM) [1], [2], Eukaryotic Linear Motif (ELM) [3], [4], and ScanSite [5], [6]. These programs all use different approaches to reduce false positive predictions.

To reduce false positive minimotif predictions, three approaches have been used in MnM [1], [2]. In frequency analysis, the complexity of minimotif sequence definitions can be used to rank-order minimotifs. A surface prediction algorithm can identify minimotifs likely to be on the surface of a protein. The third approach selects minimotifs that have conserved minimotif sequences in many species. ELM has also implemented several filters for: 1) Cell compartments, 2) Globular domains, 3) Taxonomy, and 4) Structure [3], [4], [7]. The cell compartment filter selects minimotifs where both the ligand and its target are in the same cellular compartment. The globular domain filter selects minimotifs in intrinsically disordered regions. The taxonomy filter eliminates minimotifs that are not in the same species. The structure filter selects for minimotifs that have exposure to solvent or similar secondary structural features. In ScanSite [5], [6], minimotifs are described as position-specific scoring matrices (PSSMs) that indicate the frequency of each amino acid at each position using data derived from peptide library and phage display experiments [8], [9]. ScanSite provides different stringencies of predictions.

Despite these inter-related approaches, false positives remain a concern, thus new types of filters are needed. In considering new strategies that might be used to refine minimotif predictions, we have noticed that some proteins which contain a particular domain are thought to have functions related to similar cellular processes. For example, proteins, which contain PTB or SH2 domain that binds to phospho-tyrosine containing proteins, are typically involved in signaling [10]. Likewise, BRCT domains generally bind to phosphopeptides and are often in proteins associated with DNA repair and cell cycle checkpoints [11], [12]. Therefore, we hypothesize that knowledge about molecular and cellular functions can be used to refine minimotif predictions.

The Gene Ontology (GO) database [13] contains structured information about biological processes, cellular components, and molecular functions. Proteins are associated with terms in each of these ontologies. Each ontology is structured as a directed acylic graph that maps the relationships between terms. We can take advantage of GO because it references protein accession numbers and thus, can be cross referenced to the Minimotif Miner database[1], [2].

We have built a syntactical and semantic structure for minimotifs that enables easy integration of minimotif and GO data [14]. Briefly, a minimotif is contained in a protein, which is called the ‘source protein’. A minimotif in the source protein has an action, designated ‘activity’. The protein which recognizes the minimotif to induce the activity is the ‘target protein’. For example, in [yytm in Jak2] [binds] [the SH2 domain of SHB], *yytm* is the minimotif sequence, *Jak2* is the source protein, *binds* is the activity, and *SHB* is the target protein. *SH2 domain* specifies the region where the minimotif binds the target *SHB*.

For predictions of new minimotifs, the source protein query contains multiple minimotif short sequences that may encode new activities with all of the target proteins. In this paper, the source protein and the set of putative target proteins can be mapped to cellular and molecular functions derived from the GO database to determine whether the source and target proteins share at least one common or similar cellular function and/or molecular function. This approach was first tested on the Minimotif Miner database of experimentally verified minimotifs. Analysis of several variations of the algorithm demonstrates that this approach can reduce false positive minimotif predictions in the test dataset and eliminate many predictions in a set of randomly selected query proteins.

Since there are many false positives in minimotif prediction programs, any means of selecting minimotifs with a higher probability of being true is desired. The molecular function filter does provide this advantage. Another important aspect of the filters presented in this paper is that they segregate minimotifs into groups for uses. With the cell function algorithm users can choose minimotifs for target proteins that are involved in the same cellular process or in a different cellular process. For example if the query protein is involved in cell division, one user may want to only look for minimotif predictions for other proteins involved in cell division or may want to identify predictions that are involved in other cellular processes. We have also implemented this for molecular functions as well.

Another important contribution of this paper is the novel conclusion that it may be possible to combine more than one filters to get another filter whose performance is better than that of the individual filters. In particular, we have devised two combinations. The first combination has the molecular function and the frequency score filter and the second combination has the cellular function filter and the frequency score filter. The new combination filters have much better p-values than all the component filters involved.

## Methods

### Data sources for evaluating the cellular and molecular function filter algorithms

To reduce the false positives in the minimotif predictions by MnM with cellular/molecular function information, we obtained this functional data from the GO database. We selected the GO database for this purpose because it has the largest ontologies for these functions and has relationships between functions. The GO ontology (4/09 release) has 16,698 terms and 32,719 edges for biological processes/cellular functions and 9309 terms and 9,924 edges for molecular functions. The edges for functional relationships are directed from the juxtaposed node to the larger node for two neighboring terms. Because identical proteins in the MnM and GO databases may have different accession numbers, we used an alias table to map these accession numbers to the cellular/molecular functions of each protein.

To test the effectiveness of the filter algorithms, we ideally needed to compare a dataset of verified minimotifs to known negatives. For experimentally verified minimotifs we used the Minimotif Miner 2 database (MnM 2), for which the total number of minimotifs is ∼5300 [1], [2]. 2,926 of these entries encoded minimotifs where accession numbers for both the minimotif source and target proteins were known. Of these 1,739 entries had at least one cellular function and 2,018 had at least one molecular function in the GO database. These entries were treated as the “Validated” positive dataset containing experimentally confirmed minimotifs.

We did not have access to known negative minimotifs, so we generated a dataset that will serve as “negative” interactions that are comprised of proteins that are most likely not to interact. There are ∼500,000 known protein-protein interactions for >5,000 total proteins, but if all possible pairwise interactions are considered, then the number of true minimotifs is a very small fraction of the total possible number of all interacting protein pairs. For example, if there are ∼30,000 proteins for the ∼500,000 interactions, then the total number of possible pairing is 449,985,000. Thus, it is safe to assume that choosing randomly generated protein pairings represents “negative” minimotifs. Therefore, 20,000 entries of random pairs of source proteins and target proteins were sampled. Of these pairs, 3,153 had at least one cellular function and 3,706 of the pairs had at least one molecular function in the GO dataset. These entries were used as the “Negative” datasets. *We then tested if any of these negative data points was in the positive dataset. In particular, for every minimotif in our database we generated all the (source, target) pairs. We assembled all of these pairs into a collection C. Followed by this, for every pair (A, B) in the negative dataset we checked if (A, B) was in C. None of these 20,000 pairs was in C. This is again a validation of the way we have picked the negative dataset.*

### Design and evaluation of the basic function filter algorithms

The basic function filter algorithms test whether at least one common or similar cellular/molecular function is shared by the given minimotif source protein and target protein. Given the minimotif source protein *S*, which contains the putative minimotif *p*, and a known or predicted associated target protein *T*, with the protein accession number alias table, find the list of cellular functions of each *S*, and T. Compare the two function lists to identify a set *C* of common cellular functions. The molecular function filter algorithm is identical except that it utilizes molecular functions *F*, instead of *C*.

This algorithm was applied to the above datasets for molecular and cellular functions. To evaluate the efficacy of the algorithms we used two metrics. The percentage for the experimentally verified minimotifs not removed by the filter is the *sensitivity*, while the percentage of negative minimotifs not removed by the filter is the *selectivity*. The ratio of sensitivity/selectivity is a *Discrimination Ratio (DR)* that measures the preference for verified minimotifs over that of negative minimotifs. *The choice of DR is quite natural. Please note that sensitivity is the % of true positives and selectivity is the % of false positives. Clearly, we want the true positives to be large and the false positives to be low and hence we want this ratio (i.e., DR) to be large. Ideally, if an ROC curve could be plotted, that will bring out the statistical significance nicely. For an ROC curve to be plotted there has to be an underlying parameter that changes. For some of the datasets in our analysis, there is no relevant underlying parameter that changes and hence we could not plot ROC curves for them. Also, note that the ROC curve is nothing but a plot of false positives versus true positives. In some sense we can think of the DR as a (single number) summary of the ROC curve.*

Sensitivity and selectivity both range from 0% to 100% with 100 % indicating complete recovery of the experimental minimotifs or of negative minimotifs. A *DR* above 1 indicates a favorable filtering preference, while those below 1 show worse performance in selecting the verified minimotifs rather than the negative minimotifs.

The cellular function algorithm had a sensitivity of ∼11% and a selectivity of ∼3%, with a DR of 3.9, indicating that many motifs were recovered and there was a ∼4-fold preference for retaining a verified instance over a randomly selected negative instance. The molecular function algorithm had a sensitivity of ∼29% and a selectivity of ∼13%, with a *DR* of 2.3. The test analysis shows that there is value in using molecular and cellular function filters for reducing false-positives in minimotif predictions.

### Design and evaluation of an expanded algorithm based on the function similarity

While the Cellular and Molecular function algorithms have value in reducing false positives, the structure of the GO database provided us with an opportunity to vary the stringency of function assignment and optimize these algorithms. GO contains neighborhood information of each cellular/molecular function term. The nodes are cellular function terms or molecular function terms in this case, and the edges go from the children nodes to parent nodes. So the “at least one common function” becomes “at least one similar enough function”. That is to say, the predicted target proteins are restricted to those for which at least one cellular/molecular function is similar enough to one in minimotif source protein, or the distance between at least one cellular/molecular function of the target protein and that of the source protein is small enough. We have introduced a distance threshold into the basic algorithm.

The expended algorithm works as follows: given the distance threshold *t*, for each pair of cellular/molecular functions, one from the list of *S* and the other from the list of *T* following the basic algorithm, examine their ancestors on the directed graph to see whether there exists a common ancestor function such that the total distance or the total number of edges between this ancestor and the pair of functions is smaller than or equal to the threshold *t*.

## Results

The basic algorithms used a distance threshold of 0; here we tested 5 additional distance thresholds of 1, 2, 3, 4, and 5. Results from the evaluation of the cellular function filter are shown in **Table 1**. The sensitivity showed a linear increase with node distance. The *DR* for verified minimotifs was the highest when the distance threshold was one with a 4.6-fold preference for verified minimotifs, but still showed a 3.4-fold preference for a threshold of two nodes. The sensitivity significantly increased over the basic filter by using a distance of one or two, rather than 0.

**Table 1:** Evaluation of the cellular function filter algorithm.

Distance	sensitivity	selectivity	DR
0	11%	3%	3.8
1	26%	6%	4.6
2	48%	14%	3.4
3	65%	32%	2.0
4	82%	58%	1.4
5	90%	79%	1.2

**Figure 1:**
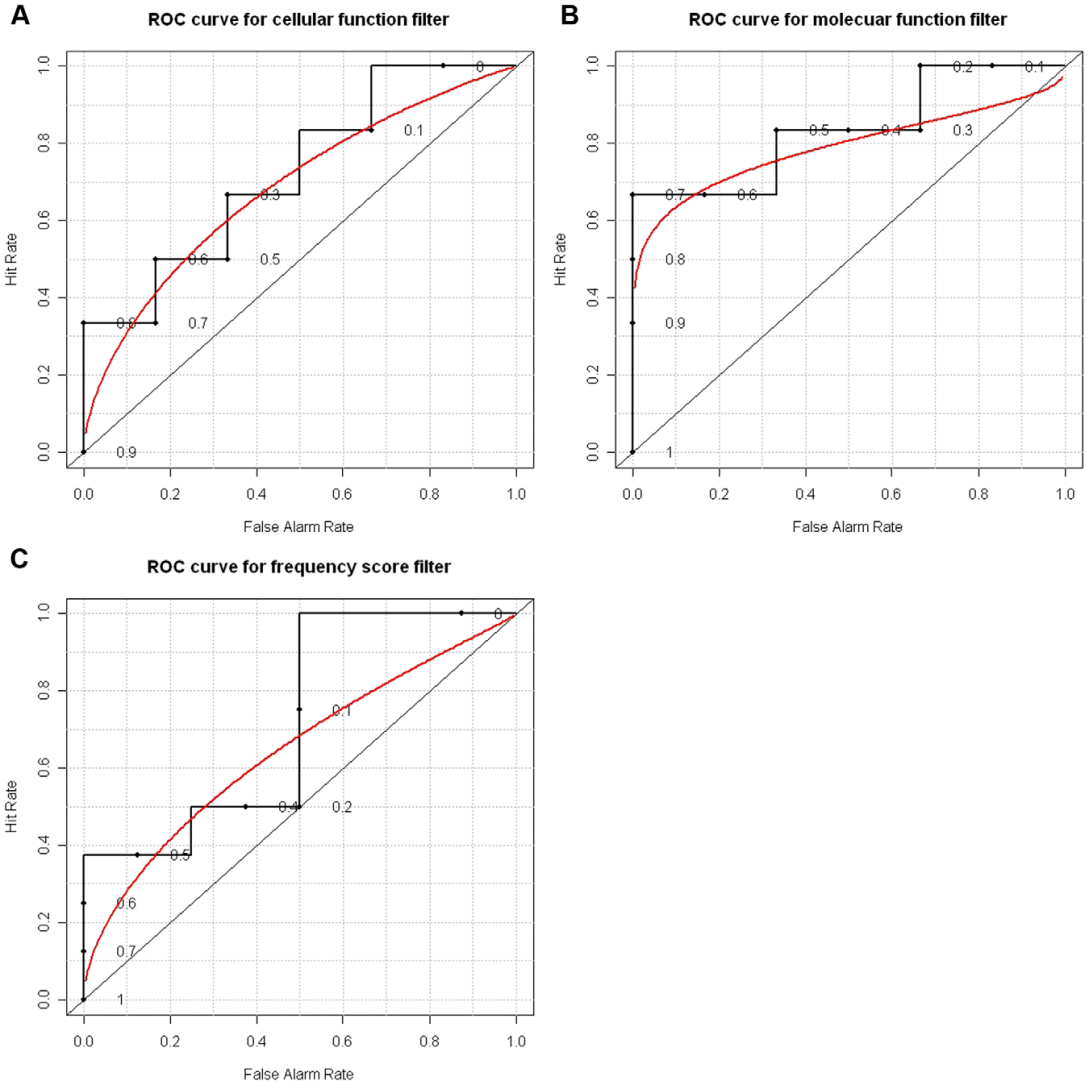
ROC curves for minimotif filters. ROC curves for the molecular (A) and cellular (B) function filters, as well as the frequency score filter are shown. Analysis was with the minimotifs in the MnM 2 database that have known molecular and cellular functions in the GO database (A,B).

To test the statistical significance of the filters we have used ROC curves and p-values. We have employed the programs of the R project [15] for this purpose. In the case of the cellular function filter, we have used the distance as the underlying parameter for plotting the ROC curve (**Figure 1A**). The area under the ROC curve is 0.7. and the p-value is 0.12. Note that p-value indicates the probability of getting the same sensitivity and selectivity results using a random predictor or filter.

Results from the evaluation of the molecular function filter are shown in **Table 2**. Again sensitivity significantly increased with distances of one or two without a major compromise in the *DR*. The molecular function algorithm is more sensitive, but less selective when compared to the cellular function filter. For the molecular function filter also, we have plotted the ROC curve with distance as the underlying parameter. **Figure 1B** shows this ROC curve. The area under the ROC curve is 0.8 and the p-value is 0.03.

**Table 2:** Evaluation of the molecular function filter algorithm.

distance	sensitivity	selectivity	DR
0	29%	12%	2.3
1	59%	21%	2.9
2	82%	35%	2.3
3	91%	50%	1.8
4	94%	61%	1.6
5	96%	72%	1.3

Both filters have value in reducing false-positives in the test datasets and stringency of predictions can be controlled by selecting distances between 0 and 3, whereas the performance of the algorithms degrades at distance values above 3. The above results indicate that the filters differentiate verified data from negative data with a good confidence and strongly suggest when predicting novel minimotifs these filters would help to decrease the number of false-positive predictions.

### A comparison with the frequency score filter

We wanted to compare the performance of the new filters with one of the already existing MnM filters, namely, the frequency score filter. To begin with we have plotted the ROC curve for the frequency score filter. This ROC curve is shown in **Figure 1C**. The area under this curve is 0.7 and the p-value is 0.08, which is similar to that of the molecular and cellular functional filters.

**Table 3** shows a comparison of the new filters with the frequency score filter on various aspects. Consistent with the ROC curves this table shows that the molecular function filter is somewhat stronger than MnM Frequency score filter in discriminating true positives from false positives. The cellular function filter is similar to the MnM frequency score filter in performance.

**Table 3:** Statistics for comparison of functional filters to the Frequency Score filter.

	Cellular Function	Molecular Function	Frequency Score	MF-FS Combination	CF-FS Combination
**Area**	0.72	0.83	0.72	0.89	0.87
**p-value**	0.12	0.03	0.08	0.002	0.0002

***Note:*** *The above results indicate that the cellular function filter has a poorer p-value than the frequency score and the molecular function filters. As a result, one has to exercise caution while employing the cellular function filter. Both the filters could be of value in clustering the motifs predicted by MnM.*

### A combination of molecular function and frequency score filters

A novel contribution of this paper is the conclusion that a combination of several filters can yield a better predictability than the individual filters. In particular, we have devised two combination filters. The first combination filter employs the molecular function and the frequency score filters. Note that these two filters are based on two different principles. The frequency score is based on the number of occurrences of the predicted motif whereas the molecular function filter is based on whether the source and target proteins share a common molecular function. Our tests of the combined filter indicate that the combined filter has a better p-value than the two individual filters.

We have employed the either-or-based combination of the molecular function filter and frequency score filter, in the expectation that the two filters can complement each other in some way, which is reasonable since they focus on different aspects and therefore the combined filter may outperform any of the two. Given a motif of some source protein, associated with its target protein, the combined filter examines whether the source and target proteins are retained by the molecular function filter, as well as whether the motif and source are retained by the frequency score filter. If either filter retains them, the combined filter retains them.

**Figure 2:**
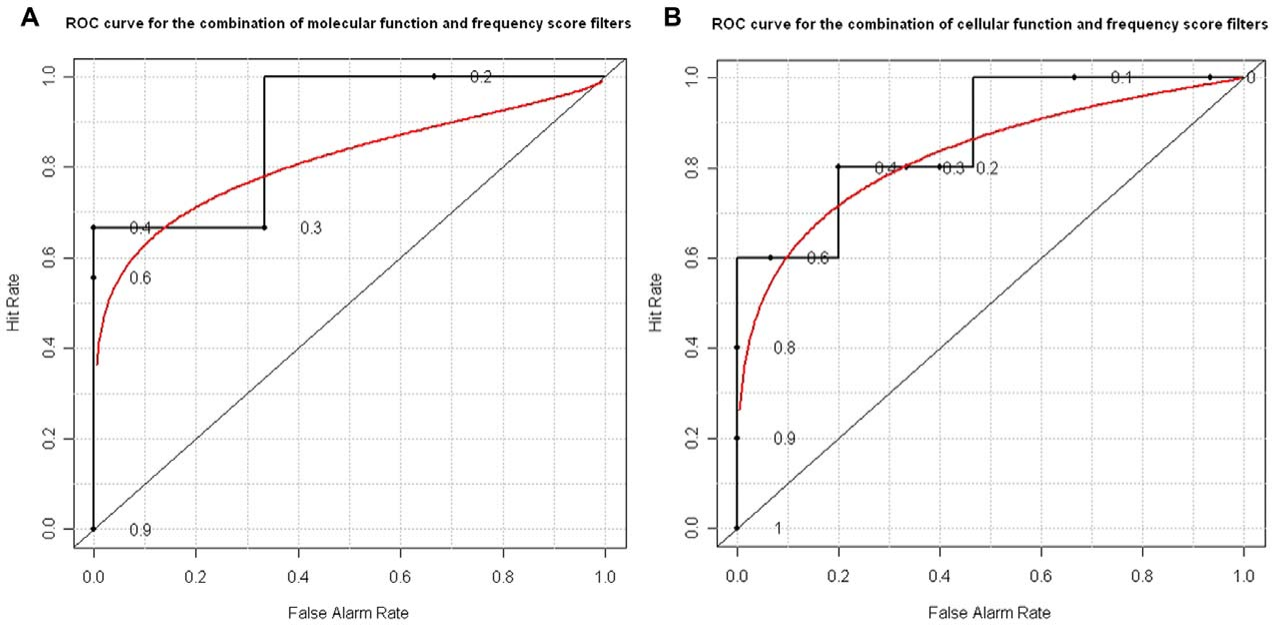
ROC curve for the combined filters. Combination of molecular function and frequency score filters (A) and combination of cellular function and frequency score filters (B) are shown. These ROC curves have been obtained by combining the two pairs of filters on an either-or basis.

This idea was tested on the same positive dataset and negative datasets. The positive datasets have already got experimentally verified entries of motif, its source protein and the associated target protein. For the negative datasets, which are 20,000 random protein pairs, we threw one of each protein pair into Minimotif Miner (MnM) [1], [2] as the source query protein and found its motif to form the triple of motif, source protein and target protein. There are totally 463, 062 such triples, of which an unknown molecular function can be found for both the source and target in GO dataset. Then three thresholds (0.02, 0.03, 0.04) for frequency score filter were picked up, together with three distances (0, 1, 2) for molecular function filter, and the nine combinations of these thresholds and distances are used as the threshold parameters of the combined filter. The prediction of the combined filter is shown in **Table 4**. To form a smooth curve, very small noises were added to the sensitivity and selectivity, which is no more than 1.463283e^−10^. The ROC curve is shown in **Figure 2A**, of which the area under the curve (AUC) is 0.89 and the p-value is 0.002, shown in **Table 3**.

**Table 4:** Evaluation of the molecular function – frequency score combined filter.

		thresholds		
		0.02	0.03	0.04
**positive data**	**distance = 0**	28%	28%	28%
	**distance = 1**	63%	63%	63%
	**distance = 2**	88%	88%	88%
**Negative data**	**distance = 0**	19%	16%	15%
	**distance = 1**	27%	24%	23%
	**distance = 2**	41%	39%	38%

### A combination of cellular function and frequency score filters

The second combination filter employs the cellular function filter and frequency score filter in the same way. Considering the cellular function filter is more stringent, five distances (0, 1, 2, 3, 4) were used, together with the same three thresholds (0.02, 0.03, 0.04) for frequency score filter. As a result, fifteen threshold parameters were formed for this combination of cellular function and frequency score filters. To smoothen the ROC curve, very small noises were also added, which is no more than 6.743894e^−11^. The prediction of this combination is shown in **Table 5** and the ROC curve is shown in **Figure 2B**, for which AUC is 0.87 and the p-value is 0.0002, shown in **Table 3**. Note that even though the frequency score filter and the cellular function filter on their own are not highly predictive, their combination is very impressive.

**Table 5:** Evaluation of the cellular function – frequency score combined filter.

		thresholds		
		0.02	0.03	0.04
**positive data**	**distance = 0**	17%	17%	17%
	**distance = 1**	44%	44%	44%
	**distance = 2**	75%	75%	75%
	**distance = 3**	88%	88%	88%
	**distance = 4**	95%	95%	95%
**negative data**	**distance = 0**	9%	6%	5%
	**distance = 1**	12%	9%	8%
	**distance = 2**	20%	17%	16%
	**distance = 3**	37%	34%	34%
	**distance = 4**	62%	60%	60%

### Implementation of cellular and molecular function filters

**Figure 3:**
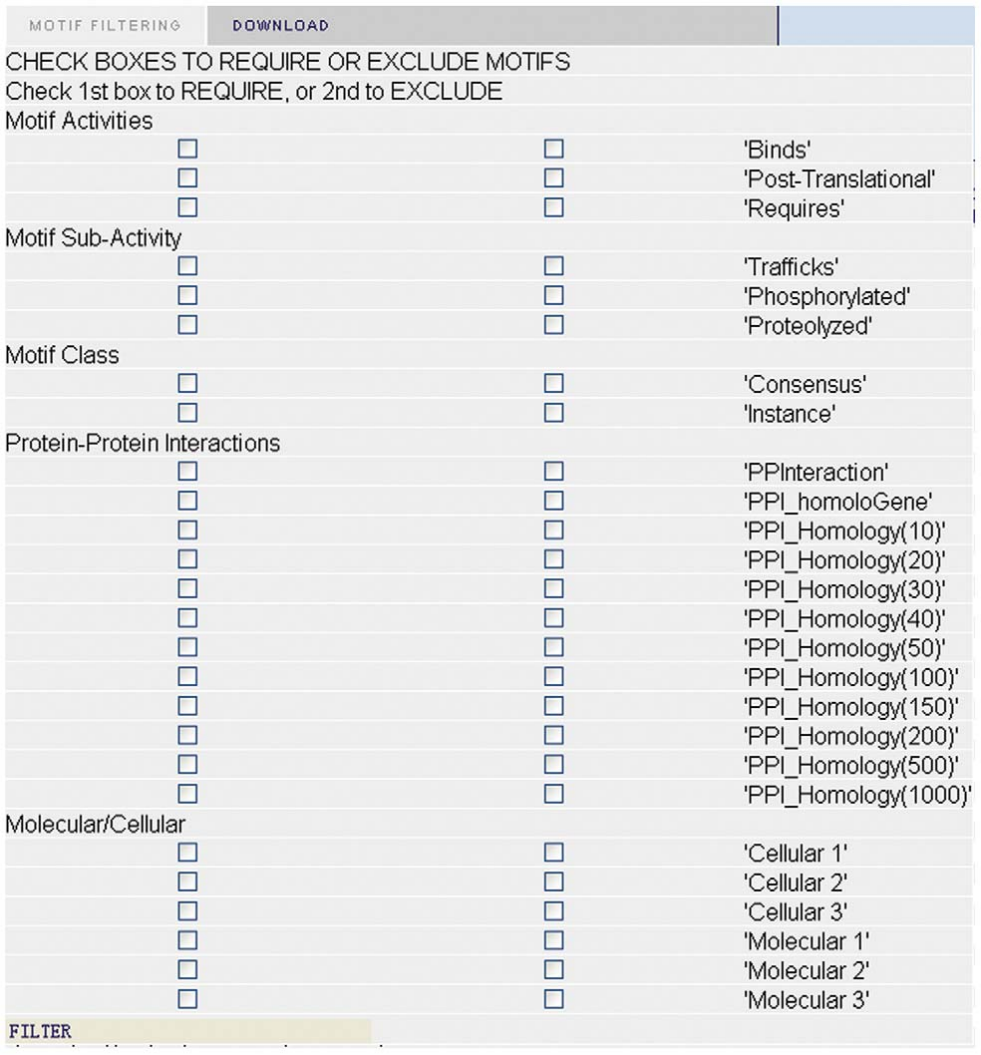
Image of the filter selector on the MnM website. All filters in this paper are now included as part of the MnM website. The option to select minimotifs that have similar or dissimilar functions is implemented.

We have implemented these new filters with the other filters on the MnM 2 website (**Figure 3**). We allow the user to vary the stringency by choosing different thresholds. We have added the results of this analysis and a description to help users interpret the results they should expect for different distance thresholds. We have also designed the implementation so that this filter can be used in combination with other MnM filters. We expect that when used in combination with other MnM filters, this will increase the specificity, but reduce the sensitivity of identifying true minimotifs. We anticipate that some users will want to look for new function of proteins and exclude minimotif predictions that are related to the known functions. Therefore, we have used a GUI checkbox that allows users to only see minimotifs that were excluded from the filter.

We wanted to examine how many predicted minimotifs were filtered by the algorithms. We ran the filter on P53, Cyclin A, and MSH2, which each have different molecular and cellular functions (22 more proteins were tested and are shown in **Supporting Information S1**). Statistics for predictions from this analysis are shown in **Table 6**. The basic Cellular function filters eliminated 90–95 percent of the target predictions, retaining only those with similar cell functions as expected. The Molecular function filter was less robust eliminating 27–48 of the minimotif predictions. Altering the GO term distance threshold also had the anticipated result where the stringency of predictions was titrated as expected.

**Table 6:** Analysis of novel queries with the cellular and molecular function filters.

			Cellular function		Molec. function	
Protein	RefSeq	Threshold	*Total	Retained	*Total	Retained
p53	NP_035770	0	64	10	67	46
p53	NP_035770	1	64	33	67	53
p53	NP_035770	2	64	52	67	63
p53	NP_035770	3	64	61	67	64
p53	NP_035770	4	64	64	67	65
p53	NP_035770	5	64	64	67	65
Cyclin A	NP_003905	0	81	3	82	38
Cyclin A	NP_003905	1	81	6	82	51
Cyclin A	NP_003905	2	81	23	82	65
Cyclin A	NP_003905	3	81	40	82	69
Cyclin A	NP_003905	4	81	64	82	72
Cyclin A	NP_003905	5	81	77	82	75
MSH2	NP_000242	0	76	8	80	25
MSH2	NP_000242	1	76	15	80	52
MSH2	NP_000242	2	76	34	80	66
MSH2	NP_000242	3	76	62	80	74
MSH2	NP_000242	4	76	73	80	76
MSH2	NP_000242	5	76	75	80	77

*Totals do not include minimotifs for which no GO terms are assigned to the proteins.

## Discussion

It is important to increase the efficiency and specificity of minimotif prediction. Many minimotif filters increase the specificity of minimotif predictions. Over time the collective use of a set of well-developed filters such as the ones we present here will lead to accurate computational tools. This is not just true for minimotifs, but for transcription factor binding sites as well. Incremental development of algorithms is a standard in computational biology.

We have reported two new filters for the elimination of false positives in minimotif predictions. Our testing results reveal that these filters are indeed effective. The use of these filters seems to be a logical approach for reducing false positives. If two proteins are involved in the same cellular or molecular function, they may be in the same or redundant pathways. However, if one contains a minimotif that is the target of another protein in the pathway, then this provides a second piece of data suggesting a functional relationship between the two proteins.

The cell function filter, eliminated 90–95% of the predictions for the 3 proteins we tested. This is the most stringent filter we have come across in the other filters designed for MnM. The frequency filter, surface prediction filter, and evolutionary conservation filters all showed a preference for filtering false positives, but not to the extent seen for the cellular function filter. The molecular function filter, while not as stringent as the cellular function filter, also performed better than previous filters implemented in MnM. This suggests that other data-driven minimotif filters used by themselves, or in combination may provide a good approach for reducing false positives. This does come at a cost, as a percentage of true minimotifs may be filtered.

We have been running Minimotif Miner for 4 years and one of the major difficulties for users is that when a list of potential target names is presented to them, most scientists do not have a knowledge-base to understand all of the different functions in the potential targets and this makes it difficult to select minimotifs for experimental testing. The new functional filters help to alleviate this problem, by restricting the predictions to those functions that are related to the query protein. In the case where a user wants to know new functions of their query, they can use the “exclude” filter, to identify only those functions that are not previously related to the query. In conclusion, the functional filters provide a valuable tool for reducing false-positive prediction of minimotifs. **Figure 3** shows a screenshot of the filter selection page in MnM.

## Supporting Information

Supporting Information S1 Supporting information document.
(0.23 MB DOC)
