# Supplementary material for: Partitioning of Minimotifs Based on Function with Improved Prediction Accuracy
Source: PLoS One. 2010 Aug 19;5(8):e12276. doi: 10.1371/journal.pone.0012276 (PMC2924378; doi:10.1371/journal.pone.0012276)
Supplement: Supporting Information S1 — Supporting information document. (0.23 MB DOC) [file pone.0012276.s001.doc]

**SUPPORTING INFORMATION**

**Table S1. Retained percentage of novel queries with the cellular function filter**

| **Protein** | **Species** | **RefSeq** | **Threshold** | | | | | |
| --- | --- | --- | --- | --- | --- | --- | --- | --- |
|  |  |  | 0 | 1 | 2 | 3 | 4 | 5 |
| IRS1 | Human | NP_005535 | 7.41% | 22.22% | 56.48% | 87.96% | 97.22% | 100.00% |
|  | Mouse | NP_034700 | 12.62% | 26.21% | 47.57% | 80.58% | 96.12% | 98.06% |
|  | Rat | NP_037101 | 5.66% | 17.92% | 39.62% | 76.42% | 95.28% | 98.11% |
| INPP5A | Human | NP_005530 | 0.00% | 17.74% | 43.55% | 77.42% | 82.26% | 95.16% |
| GP1BA | Human | NP_000164 | 5.48% | 24.66% | 68.49% | 86.30% | 94.52% | 97.26% |
|  | Mouse | NP_034456 | 5.97% | 7.46% | 32.84% | 65.67% | 83.58% | 97.01% |
| GAP43 | Rat | NP_058891 | 0.00% | 0.00% | 9.68% | 45.16% | 67.74% | 83.87% |
|  | Human | NP_002036 | 0.00% | 5.88% | 52.94% | 79.41% | 94.12% | 100.00% |
|  | Mouse | NP_032109 | 0.00% | 0.00% | 35.48% | 61.29% | 87.10% | 100.00% |
| CD2 | Human | NP_001758 | 5.17% | 31.03% | 68.97% | 75.86% | 84.48% | 93.10% |
|  | Mouse | NP_038514 | 3.08% | 3.08% | 27.69% | 60.00% | 80.00% | 96.92% |
|  | Rat | NP_036962 | 1.79% | 1.79% | 33.93% | 57.14% | 73.21% | 96.43% |
| DYRK1A | Rat | NP_036923 | 5.75% | 27.59% | 28.74% | 37.93% | 39.08% | 60.92% |
|  | Human | NP_001387 | 5.81% | 29.07% | 33.72% | 45.35% | 58.14% | 72.09% |
|  | Mouse | NP_001106860 | 25.29% | 28.74% | 35.63% | 39.08% | 56.32% | 65.52% |
| TRPM6 | Mouse | NP_700466 | 22.61% | 28.70% | 38.26% | 42.61% | 60.00% | 82.61% |
|  | Human | NP_060132 | 23.93% | 29.91% | 38.46% | 42.74% | 60.68% | 82.91% |
| DNASE1L2 | Human | NP_001365 | 0.00% | 0.00% | 13.46% | 28.85% | 67.31% | 100.00% |
|  | Mouse | NP_079994 | 0.00% | 0.00% | 5.13% | 20.51% | 66.67% | 100.00% |
| CELSR3 | Human | NP_001398 | 0.73% | 7.30% | 16.06% | 80.29% | 90.51% | 97.08% |
|  | Mouse | NP_536685 | 0.00% | 6.06% | 10.61% | 47.73% | 88.64% | 98.48% |
|  | Rat | NP_112610 | 0.77% | 7.69% | 17.69% | 81.54% | 92.31% | 98.46% |
| SLC22A3 | Human | NP_068812 | 0.00% | 2.56% | 8.97% | 47.44% | 71.79% | 91.03% |
|  | Mouse | NP_035525 | 0.00% | 0.00% | 7.35% | 41.18% | 75.00% | 94.12% |
|  | Rat | NP_062103 | 0.00% | 0.00% | 4.35% | 15.94% | 37.68% | 37.68% |
| NXPH3 | Human | NP_009156 | 0.00% | 4.62% | 7.69% | 30.77% | 61.54% | 72.31% |
| NR5A1 | Human | NP_004950 | 16.42% | 34.33% | 68.66% | 91.04% | 100.00% | 100.00% |
|  | Mouse | NP_620639 | 0.00% | 8.06% | 22.58% | 74.19% | 96.77% | 98.39% |
|  | Rat | XP_001054966 | 0.00% | 1.61% | 9.68% | 20.97% | 67.74% | 98.39% |
| RP1L1 | Human | NP_849188 | 8.99% | 28.09% | 34.83% | 68.54% | 89.89% | 97.75% |
|  | Mouse | NP_666358 | 6.98% | 20.93% | 41.86% | 86.05% | 91.86% | 96.51% |
| USP8 | Mouse | NP_062703 | 6.19% | 8.85% | 19.47% | 45.13% | 76.99% | 91.15% |
|  | Fly | NP_650948 | 2.50% | 3.75% | 3.75% | 6.25% | 20.00% | 63.75% |
| COBRA1 | Human | NP_056271 | 2.99% | 2.99% | 13.43% | 26.87% | 80.60% | 98.51% |
|  | Mouse | NP_067368 | 4.29% | 4.29% | 14.29% | 27.14% | 78.57% | 98.57% |
| MCOLN1 | Mouse | NP_444407 | 1.09% | 1.09% | 1.09% | 1.09% | 3.26% | 26.09% |
|  | Human | NP_065394 | 1.10% | 1.10% | 1.10% | 1.10% | 3.30% | 24.18% |
| ERBB3 | Human | NP_001973 | 26.83% | 64.23% | 91.06% | 95.12% | 99.19% | 100.00% |
|  | Mouse | NP_034283 | 25.64% | 41.88% | 64.10% | 77.78% | 96.58% | 100.00% |
|  | Rat | NP_058914 | 31.36% | 46.61% | 64.41% | 94.92% | 98.31% | 100.00% |
| DLGAP1 | Rat | NP_075235 | 6.82% | 6.82% | 23.86% | 69.32% | 84.09% | 93.18% |
|  | Human | NP_001003809 | 0.00% | 12.68% | 14.08% | 33.80% | 81.69% | 92.96% |
|  | Mouse | NP_808307 | 0.00% | 10.34% | 11.49% | 35.63% | 83.91% | 93.10% |
| TYK2 | Human | NP_003322 | 24.72% | 31.46% | 41.57% | 44.94% | 60.67% | 68.54% |
|  | Mouse | NP_061263 | 25.84% | 30.34% | 43.82% | 47.19% | 60.67% | 67.42% |
| AGTR1 | Human | NP_000676 | 10.29% | 27.94% | 76.47% | 91.18% | 100.00% | 100.00% |
|  | Mouse | NP_796296 | 5.80% | 10.14% | 33.33% | 79.71% | 92.75% | 95.65% |
|  | Rat | NP_112247 | 1.49% | 7.46% | 37.31% | 73.13% | 91.04% | 100.00% |
| IL1B | Human | NP_000567 | 6.25% | 46.88% | 90.63% | 96.88% | 100.00% | 100.00% |
|  | Mouse | NP_032387 | 3.33% | 13.33% | 73.33% | 93.33% | 96.67% | 100.00% |

**Table S2. Retained percentage of novel queries with the molelucar function filter**

| **Protein** | **Species** | **RefSeq** | **Threshold** | | | | | |
| --- | --- | --- | --- | --- | --- | --- | --- | --- |
|  |  |  | 0 | 1 | 2 | 3 | 4 | 5 |
| IRS1 | Human | NP_005535 | 8.11% | 13.51% | 81.98% | 86.49% | 88.29% | 90.09% |
|  | Mouse | NP_034700 | 15.09% | 33.02% | 72.64% | 86.79% | 88.68% | 92.45% |
|  | Rat | NP_037101 | 8.26% | 12.84% | 64.22% | 90.83% | 94.50% | 96.33% |
| INPP5A | Human | NP_005530 | 0.00% | 1.59% | 47.62% | 79.37% | 85.71% | 92.06% |
|  | Mouse | NP_898967 | 0.00% | 0.00% | 1.49% | 8.96% | 11.94% | 17.91% |
|  | Fly | NP_733069 | 0.00% | 0.00% | 1.92% | 7.69% | 9.62% | 13.46% |
| GP1BA | Human | NP_000164 | 44.16% | 66.23% | 81.82% | 84.42% | 85.71% | 93.51% |
|  | Mouse | NP_034456 | 47.83% | 69.57% | 84.06% | 85.51% | 86.96% | 89.86% |
| GAP43 | Rat | NP_058891 | 0.00% | 39.39% | 63.64% | 72.73% | 81.82% | 84.85% |
|  | Human | NP_002036 | 2.63% | 36.84% | 60.53% | 65.79% | 73.68% | 76.32% |
|  | Mouse | NP_032109 | 0.00% | 39.39% | 63.64% | 72.73% | 81.82% | 84.85% |
| CD2 | Human | NP_001758 | 42.62% | 63.93% | 85.25% | 85.25% | 88.52% | 95.08% |
|  | Mouse | NP_038514 | 0.00% | 7.58% | 69.70% | 84.85% | 89.39% | 90.91% |
|  | Rat | NP_036962 | 35.71% | 58.93% | 78.57% | 82.14% | 89.29% | 92.86% |
| DYRK1A | Rat | NP_036923 | 31.87% | 60.44% | 79.12% | 89.01% | 93.41% | 97.80% |
|  | Human | NP_001387 | 32.22% | 60.00% | 78.89% | 88.89% | 93.33% | 97.78% |
|  | Mouse | NP_001106860 | 60.44% | 73.63% | 86.81% | 91.21% | 93.41% | 98.90% |
| TRPM6 | Mouse | NP_700466 | 44.63% | 44.63% | 47.93% | 49.59% | 94.21% | 97.52% |
|  | Human | NP_060132 | 46.72% | 46.72% | 49.18% | 50.00% | 94.26% | 98.36% |
| NBEA | Fly | NP_001036261 | 0.00% | 48.46% | 78.46% | 87.69% | 91.54% | 96.15% |
| DNASE1L2 | Human | NP_001365 | 37.74% | 62.26% | 86.79% | 86.79% | 88.68% | 92.45% |
|  | Mouse | NP_079994 | 0.00% | 0.00% | 0.00% | 0.00% | 2.50% | 45.00% |
| BAZ2A | Human | NP_038477 | 49.07% | 67.59% | 86.11% | 88.89% | 88.89% | 89.81% |
| CELSR3 | Human | NP_001398 | 49.29% | 75.00% | 88.57% | 90.00% | 90.71% | 94.29% |
|  | Mouse | NP_536685 | 8.09% | 8.09% | 25.00% | 34.56% | 89.71% | 93.38% |
|  | Rat | NP_112610 | 8.27% | 8.27% | 25.56% | 35.34% | 89.47% | 93.23% |
| SLC22A3 | Human | NP_068812 | 49.38% | 69.14% | 82.72% | 86.42% | 87.65% | 91.36% |
|  | Mouse | NP_035525 | 44.29% | 68.57% | 81.43% | 87.14% | 88.57% | 91.43% |
|  | Rat | NP_062103 | 46.48% | 70.42% | 83.10% | 87.32% | 88.73% | 91.55% |
| NXPH3 | Human | NP_009156 | 3.03% | 51.52% | 71.21% | 77.27% | 83.33% | 84.85% |
|  | Mouse | NP_570928 | 2.99% | 50.75% | 73.13% | 79.10% | 85.07% | 86.57% |
| NR5A1 | Human | NP_004950 | 50.00% | 70.59% | 85.29% | 89.71% | 89.71% | 94.12% |
|  | Mouse | NP_620639 | 49.21% | 71.43% | 87.30% | 92.06% | 92.06% | 96.83% |
|  | Rat | XP_001054966 | 17.46% | 23.81% | 60.32% | 87.30% | 92.06% | 96.83% |
| USP8 | Mouse | NP_062703 | 43.59% | 64.96% | 81.20% | 84.62% | 89.74% | 94.02% |
|  | Fly | NP_650948 | 42.68% | 64.63% | 82.93% | 85.37% | 87.80% | 93.90% |
| COBRA1 | Human | NP_056271 | 44.29% | 70.00% | 82.86% | 87.14% | 88.57% | 91.43% |
|  | Mouse | NP_067368 | 43.84% | 68.49% | 82.19% | 86.30% | 87.67% | 90.41% |
| CSF2RA | Human | NP_006131 | 0.00% | 0.00% | 47.95% | 91.78% | 94.52% | 97.26% |
|  | Mouse | NP_034100 | 0.00% | 0.00% | 37.74% | 83.02% | 86.79% | 94.34% |
| MCOLN1 | Mouse | NP_444407 | 5.32% | 5.32% | 19.15% | 23.40% | 89.36% | 89.36% |
|  | Human | NP_065394 | 5.38% | 5.38% | 18.28% | 18.28% | 88.17% | 89.25% |
| ERBB3 | Human | NP_001973 | 28.91% | 60.16% | 87.50% | 96.09% | 97.66% | 98.44% |
|  | Mouse | NP_034283 | 30.25% | 30.25% | 30.25% | 45.38% | 50.42% | 57.14% |
|  | Rat | NP_058914 | 30.83% | 30.83% | 30.83% | 46.67% | 51.67% | 58.33% |
| DLGAP1 | Rat | NP_075235 | 41.76% | 64.84% | 81.32% | 83.52% | 85.71% | 87.91% |
|  | Human | NP_001003809 | 40.28% | 65.28% | 79.17% | 81.94% | 84.72% | 87.50% |
| TYK2 | Human | NP_003322 | 32.61% | 32.61% | 38.04% | 83.70% | 92.39% | 94.57% |
|  | Mouse | NP_061263 | 66.30% | 78.26% | 91.30% | 93.48% | 93.48% | 96.74% |
| AGTR1 | Human | NP_000676 | 0.00% | 1.45% | 65.22% | 89.86% | 95.65% | 97.10% |
|  | Mouse | NP_796296 | 47.83% | 66.67% | 84.06% | 88.41% | 91.30% | 95.65% |
|  | Rat | NP_112247 | 0.00% | 51.47% | 76.47% | 86.76% | 92.65% | 94.12% |
| IL1B | Human | NP_000567 | 0.00% | 0.00% | 50.00% | 87.50% | 93.75% | 96.88% |
|  | Mouse | NP_032387 | 0.00% | 0.00% | 26.67% | 73.33% | 86.67% | 90.00% |
